# Analysis of the coupled and coordinated development of sports and tourism industries and the driving factors

**DOI:** 10.1038/s41598-023-44025-6

**Published:** 2023-10-04

**Authors:** Maoteng Cheng, Lu Zhang, Danyi Li

**Affiliations:** 1 Center of Sports Industry Research, Shanxi University of Finance and Economics, Taiyuan, 030006 Shanxi China; 2 School of Physical Education, Ningbo University, Ningbo, 315211 Zhejiang China

**Keywords:** Environmental sciences, Environmental social sciences

## Abstract

The Yellow River Basin has a wealth of tourism resources, a long history of folk sports, and strong legislative support, all of which are advantages for the growth of the sports tourism sector. This study constructs an evaluation index system based on panel data from nine provinces and regions in the Yellow River Basin from 2011 to 2020, and then measures the development index and analyzes the driving factors using the entropy method, coupling coordination degree model, kernel density estimation method, and grey correlation analysis method. The findings demonstrate that (1) the Yellow River Basin's overall level of growth in the sports and tourist sectors exhibits a consistent and upward trend and that Shandong and Henan provinces clearly have an edge over other provinces in terms of development. (2) The geographical development pattern was lower Yellow River > upper Yellow River > middle Yellow River, and the coupling coordination status shifted from slight disorder to good coordination. (3) There is a close connection between sports tourism and variables including population density, financial prowess, and infrastructure growth. The employment population index has the smallest link with invention patent authorization. The macro-policy framework should therefore be strengthened in the future, the sports tourism infrastructure should be improved, and the Yellow River basin's unique sports tourism resources should be fully utilized in order to increase brand influence. In addition, new sports tourism products should be developed in response to market demand and consumer preferences, and enterprise innovation and research and development efforts should be increased in order to achieve high-quality development transformation.

## Introduction

The sports tourism business, which is growing at an average annual growth rate of more than 14% worldwide, is one particularly remarkable illustration of how the industrial integration tendency has evolved and risen rapidly in recent years. The combination of "sports" and "tourism" led to the emergence of a new leisure and entertainment sector, with China's sports tourism industry and tourism industry accounting for around 5% of the total, compared to 25% in industrialized nations. In a broad sense, the rationalization and modernization of industrial structure has currently become the primary objective of current industrial adjustment due to the advent of the national fitness wave. It is a new cross-industry based on sports resources and in the form of tourism activities, as well as a sunrise industry and a green industry with service as its primary focus. The integration and development of the sports industry and the tourism industry. The continuation of traditional sports and the responsible growth and complete protection of destination tourism are achieved through the integration of sport and tourism. This helps to promote a development environment where humans and nature live in harmony and reduces carbon emissions and resource waste. In a narrow sense, more and more sports and tourism consumers are no longer satisfied with the traditional consumption modes of sports and tourism, but pursue more participatory and more valuable sports and tourism modes. This has promoted the integrated development of the sports tourism industry to a certain extent^1^. The Yellow River basin has rich sports tourism resources, the ethnic folk culture in the region has a long history, complex terrain, diverse landforms, suitable for a variety of outdoor sports tourism activities, such as outdoor adventure, rafting, skiing, wilderness camping and cliff downhill and other characteristic activities. As a new economic growth point, the characteristic sports tourism activities have gradually been given great importance by the government. The Yellow River Basin's provinces and regions have progressively released pertinent guidelines to support the growth of sports tourism as a whole, including the integration of the sports and health industries, cultural tourism, education, and training. The development of sports media, sports exhibitions, and other related formats should be encouraged. Planning and building sports tourism scenic spots should also be strengthened. Sports tourism routes should be developed, and effective strategies for the integrated development of sports tourism should be looked into. In order to exert economic value, social benefits, and ecological protection, studying and examining the coupling and coordinated development level of sports tourism is of great significance. It also enlightens the development mode of sports tourism in developing countries, represented by China.

Specifically, sports tourism, as an important branch of the sports industry, is actively influencing residents to form new consumption concepts^2^. On the one hand, the sports tourism sector clearly plays an innovative, coordinating, environmentally conscious, open, and sharing role under the guidance of new development ideas, which is beneficial for sharing technical innovation and accomplishments in the process of integrating sports tourism. To strengthen the industrial structure and enable the city attract top talent and thrive, more tertiary industry should be developed. These factors include improved economic effectiveness, attracting top talent, and promoting societal peace. They make up the ideal circulation system for the sports tourism sector when combined. As two key industrial sectors with distinct internal influencing variables and constituent factors, sports and tourism, it is necessary to utilize the coupling coordination degree model to assess the degree of system interaction. On the other hand, from the standpoint of regional development, the sports tourism industry may experience major variances in the overall level of development because of the various policy direction, economic level, and industrial foundation in the time and space sequence. The entire size of the Chinese sports industry was $413.59 billion in 2019, up from 239.98 billion yuan in 2015, with an average annual growth rate of 14.58% over the previous five years. It is important to note that in 2020, the COVID-19 pandemic had a significant negative impact on the development environment and caused a 7% decline in the size of China's sports business. It is clear that the social environment has a significant impact on how the sports tourism sector is developing^3,4^. In order to explore the influencing and driving relationship between various variables on the coupling coordination of sports tourism, grey correlation analysis is required.

In order to analyze the degree of coupling coordination and the impact of driving factors on the sports tourism industry over the past 10 years, this paper empirically studies panel data of nine provinces and regions in the Yellow River Basin of China from 2011 to 2020. This is done on the basis of the research background mentioned above. The coupling coordination degree model and gray correlation analysis were employed as research techniques. The following are the study's research questions: the goals of this study are to: (1) ascertain changes in the level of development of the sports and tourism industries in the Yellow River Basin over the past ten years; (2) examine the pattern of evolution of the coupled coordination level of sports tourism in the Yellow River Basin over the past ten years and the current state of regional differences; and (3) evaluate the magnitude of the influence of various variable factors on the degree of association of the two industries.

This essay's remaining sections are organized as follows: the research methodology, data sources, and index system construction are presented in "Literature review", the literature review is presented in “Study design”, and the development level and coupling coordination status of the sports and tourism industries in the Yellow River Basin are examined based on the empirical findings in "Measurement and coupling evaluation of sports and tourism industry in Yellow River Basin". In "Correlation measurement and driving factors analysis of sports and tourism industry in Yellow River Basin", the measurement and correlation analysis of gray correlation are described after the choice of the sports and tourism industry correlation indexes.

## Literature review

Academic research has steadily shifted its attention to sports tourism since the turn of the twenty-first century. This report discovers that the current research on sports tourism mostly includes the following features by conducting a subject search for "sports tourism" in the core collection database of the Web of Science and CNKI database. Interdisciplinary integration has gained popularity among academic disciplines, and the viewpoints of social science, education, management, political economics, and other fields continue to support the development of the sports tourism research infrastructure. Theoretical analysis and mathematical statistics are utilized as research methods to carry out thorough and organized research on sports tourism. The research results have covered the impact of the sports tourism economy, society and residents' perception^5–7^, the large-scale development of sports tourism events^8,9^, the mass participation of sports tourism and the satisfaction of sports tourism events^10,11^, research on sports tourism nostalgia and marketing^12–15^,research on sports tourism heritage^16^. In the research content of sports tourism, the concept of sports tourism is defined, and it is considered that sports tourism is a vacation activity involving sports activities for participants or visitors^17^. Furthermore, scholars have made a more detailed definition of regional sports tourism, believing that the differentiated development of sports tourism products in a certain region can meet the individual needs of the market by relying on local sports resources or tourism services^18^. The definition's breadth eventually shifts to a micro perspective, and it increasingly focuses on the market economic worth of sports tourism. As a result, researchers keep researching the particular practice of sports tourism. For instance, when it comes to rural sports tourism, academics feel that it is the culmination of a number of elements and variables that can draw in sports tourists, facilitate the conduct of sports tourism activities in rural regions, and be utilized by tourism to achieve positive social, economic, and environmental outcomes^19^. As a result, farmers' incomes may rise, jobs may be created, rural economies may flourish, regional industrial structures may be optimized, the gap between urban and country areas may be closed, and the rural economy may develop more sustainably^20^. Scholars have, however, compiled and examined this as they gradually discovered, over the course of these practical analyses, that the integration mode of sports tourism has various development models in addition to regional resource disparities. According to academics, the development of the sports tourism industry is largely dependent on the integration of the government, market, organization, management, technology, and talent. This creates a multi-subject collaborative innovation pattern of "industry, school, government, research, and use" and creates a new system of value chains, production chains, and innovation chains for industrial integration^21^. In terms of restructuring, experts believe that sports tourism can be restructured to create a new industry through the interrelated products or services of the two, with events serving as the platform to create event sports tourism, exhibitions serving as the carrier to create exhibition sports tourism, and festivals serving as the connotation of festival culture to create festival sports tourism. This will enable the realization of the diversification of sports tourism products to encourage sports consumption^22^. The experience economy, which is primarily driven by consumer demand, has been noted by academics. They have suggested expanding the sports industry's industrial chain, which is dominated by competition performances, the consumer travel experience, and the defining services of shopping, entertainment, and tourism culture^23^. According to its unique industrial characteristics, sports tourism engages in two-way penetration that neither modifies the original industrial form nor creates new business models^24^. The most typical integration mode, which is a primary development mode, is also the one with the least amount of integration^25^.

This study employs the search words "industrial integration" and "sports and tourism coupling" to carry out further research because scholars in the field of sports and tourism integration have produced a wealth of research results. The trend of industrial integration was promoted in the 1960s against the backdrop of the third wave of scientific and technological revolution, and scholars have primarily used the basic theories such as the theory of industrial integration, synergy theory, game theory, and other basic theories to explain the basis and in order to explain the deep-level innovation and development of sports and tourism. For instance, according to academics, the penetration and integration of technology, resources, markets, products, and other components to create new sectors and new business models is at the heart of the integration of sports tourism^26^. Scholars then proposed "digital integration" to replace "technology integration" in response to the wave of information technology. Digital technology drives the advancement of industrial integration, which is driven by the organization and service integration of the value creation process^27^. As a result, the sports tourism industry's shared technological resource base is one of the fundamental prerequisites for the development of industrial integration when analyzing the reasons of sports tourism sector integration^28^. To the new concept of integrating the sports tourism business, new components like digital technology and artificial intelligence must be added. Following this, scholars have engaged in a significant amount of empirical study and inquiry in the current context of industrial integration motivated by digital integration, yielding positive research findings^29,30^.

Some research results have been produced in the research of regional sports tourism. Taking the Yellow River Basin as an example, scholars have carried out detailed research on sports tourism policy^31^, ecological protection of sports tourism^32^, development of fine sports tourism projects^33^ and sustainable development of sports tourism^34^ in the Yellow River Basin. According to academics, the state presently provides the Yellow River Basin with ecological protection and a high-quality development strategy. This advantageous policy is both a policy support for attaining high-quality development of sports tourism and a significant historical opportunity for the transformation and growth of the industry. A quality project for sports tourism is crucial for guiding and advancing the industry's growth, as well as for enhancing and broadening the sports tourist market. The Yellow River basin's high-quality growth of sports tourism can be supported by making use of and exercising the benefits of the Yellow River's sporting tourism resources and by constructing sporting tourism demonstration projects. Researchers primarily advocate the study of sports tourism in the Yellow River Basin through inductive reasoning and field research. The research's subject matter is often not too complicated.

As a result, industry attention has recently shifted to research on the integration of sports tourism, both from the perspective of government policy and academic theoretical debate. Sports tourism's integration condition, necessity, and development mode have all been thoroughly examined in recent research, but the systematic study has not gone into enough detail or for long enough. The principal limitations are as follows: first, the expansion of the sports tourism business lags behind the creation and measurement of industry evaluation indicators in terms of research content^35^. Second, the research scale rarely involves the construction and measurement of regional industry evaluation indicators^36^. Thirdly, little research has been done on what motivates the sports and tourist industries^37,38^. Based on this, the paper's research focuses on the sports and tourist sectors in nine provinces and areas of the Yellow River Basin. In order to construct the industry's development assessment index system, it incorporates the input–output concept. It also looks into the industry's dynamic evolution process and how its driving elements relate to its form, coupling, and coordination levels. Based on the existing literature, the contribution of this paper is as follows: first, an input–output concept-based industrial development evaluation index system is constructed, and the entropy approach is utilized to objectively weight the indexes and ascertain the overall development level of the sports and tourist sectors. Second, using kernel density estimation, which also determines changes in the coupling coordination level between the sports and tourism industries over the previous ten years, the evolution characteristics of the sports tourism industry in the upper, middle, and lower reaches of the Yellow River basin are examined. Third, the introduction of the grey correlation analysis method measuring the indexes and coupling coordination level of the Yellow River sports tourism industry is the coupling, and at the same time, high quality, sustainable, and innovative development countermeasures and suggestions are being made for the Yellow River sports tourism industry in developing countries, including China.

## Study design

### Overview of the study area

**Figure 1:**
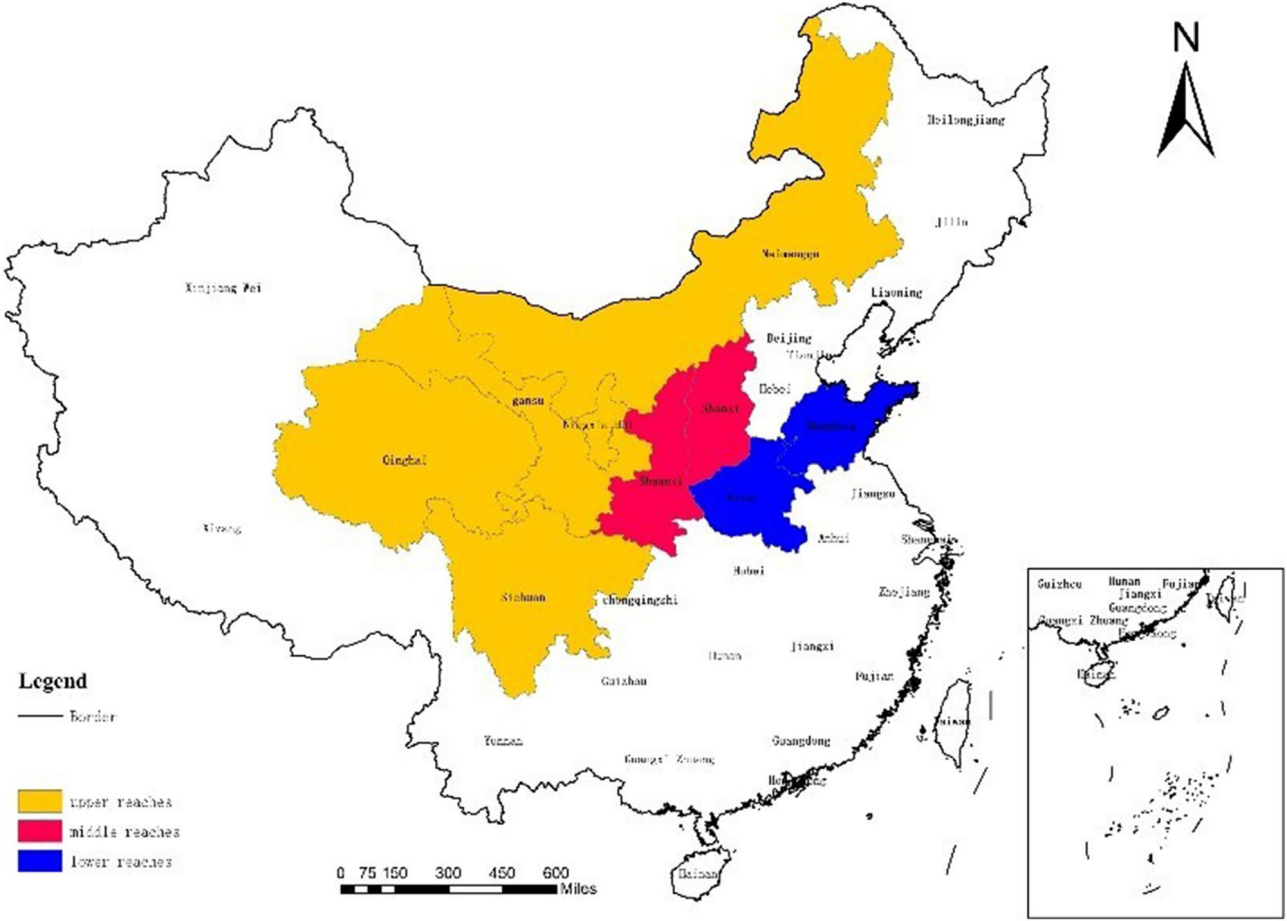
Nine provinces and regions in the Yellow River Basin. Source: Based on the standard map service website of the Ministry of Natural Resources, the approval number is GS (2020)4630. The base map boundary is not modified.

The Yellow River, sometimes referred to as China's "Mother River", is the second-largest river in China and the fifth-largest river in the world in terms of studied area. Geographically, the Yellow River basin has an area of 795,000 square kilometers and extends 5464 km from east to west. It is located in China's northern region. The Yellow River flows through nine provinces: Qinghai, Sichuan, Gansu, Ningxia, Inner Mongolia (upstream part); Shaanxi, Shanxi (midstream part); Henan, Shandong (downstream part); and finally, it empties into the mouth of the Bohai Sea (Fig. 1). Tourists can find a ton of natural features in the Yellow River valley, including as mountains, rivers, waterfalls, meadows, snow and ice fields, and other geomorphic marvels. Twenty of these are found in the Yellow River basin, including Mount Tai in Shandong, the Jiuzhaigou plateau in northwest Sichuan, and the Mogao Caves in Dunhuang. There are 84 national 5A scenic areas in total, covering 27.45% of the nation. There are 47 demonstration locations for all-area tourism and 649 intangible cultural heritage objects at the national level^39^. The number and caliber of sporting events in the Yellow River Basin are among the best in China, with the Tour of Qinghai Lake International Road Cycling Race, which will be held 21 times by 2022, the Taiyuan International Marathon, which features double gold standard competitions, the Around Binzhou Yellow River scenery belt International Road Cycling Race, and others serving as representative events. Each year, these events draw more than 26 million domestic and international sports tourism spectators. The Yellow River Basin's GDP, measured in US dollars, was 3.56 trillion in 2020, or 25.01% of the entire nation. The output value of the tertiary industry was 1.82 trillion US dollars out of this total, or 23.45% of the entire country. The tourism industry in the Yellow River Basin will bring in 0.77 trillion US dollars in total income in 2020, while the country's sports industry will bring in 95.457 trillion US dollars, or 23.08% of the total, according to industrial segmentation^40^. As can be seen, despite having a relatively low degree of economic development in comparison to the rest of the country, the Yellow River Basin has a strong industrial base and abundant sporting and tourism facilities. This is especially true in light of the present national plan, which strongly emphasizes the preservation of the biological environment in the Yellow River Basin and the promotion of high-quality industrial development. The potential deep integration of the sports and tourism industries holds a lot of promise for the Yellow River Basin^41,42^.

### The research methods

#### The entropy value method

Numerous research findings on the numerous industrial integration measuring approaches are available in the academic world. The expert survey approach, the AHP method, the fuzzy evaluation method, the entropy method, etc. are only a few examples of the numerous methods available to generate weights. Expert scoring, sometimes referred to as questionnaire surveys or the subjective assignment technique, is the main method used to evaluate the expert survey method and the AHP method. Such methods could easily lead to a disparity in measurement results and an excessive subjective factor intervention^43^. The data information concentration notion, which demands a lot of data, is the basis of both the factor analysis approach and the fuzzy assessment principle. The degree of data information variation is used in the entropy approach to weight the index. This method is suited for data that exhibits volatility, and data volatility will be utilized to determine the degree of index dispersion. The influence of an assessment index on an overall evaluation grows with the level of dispersion^44,45^. The entropy approach is more accurate and objective in light of the aforementioned factors, and the produced data may successfully depict changes in the studied object's trend. In order to determine how far the sports and tourist industries have progressed, the entropy approach is applied. The exact steps are as follows:

First, because each indication vary in nature and magnitude, it is challenging to directly compare and synthesize several indicators for the goal of normalization. To ensure the validity of the results and harmonize the comparison criteria, the original variables must be normalized prior to data analysis. Relevant research standardize evaluation indicators using a variety of methods, including standard deviation, the extreme value approach, and others. Based on an effective and scientific data processing strategy, the extreme value approach is selected as the normalizing method. This study adds 0.0001 to the normalized data overall to assure data processing integrity because all data are between [0, 1] after the extreme value technique is utilized to process the data.

Second, after normalizing the original indicator data, find the contribution of year i of the jth indicator to the indicator.

1 $${P}_{ij}=\frac{{X}_{ij}}{\sum_{i=1}^{n}{X}_{ij}}$$

Third, find the entropy value of the jth indicator.

2 $${e}_{j}=-\frac{1}{\mathit{ln}n}\sum_{i=1}^{n}{P}_{ij}\mathit{ln}{P}_{ij} 0\le {e}_{ij}\le 1$$

Since the study sample data is for the period 2011–2020, n = 10 and $-\frac{1}{\mathit{ln}n}$= $-0.4343$ .

Fourth, calculate the variability factor.

3 $${g}_{j}=1-{e}_{j}$$

Fifth, calculate the evaluation index weights ${w}_{j}$.

4 $${w}_{j}=\frac{{g}_{j}}{\sum_{i=1}^{m}{g}_{j}}$$

Sixth, find the combined score for sports and tourism.

5 $${ U}_{i}={\sum }_{i=1}^{m}{w}_{j}{x}_{ij}$$

#### Coupling coordination degree model

It is possible to see how the mutual influence and promotion of the sports and tourist industries have developed over the past few years using the coupled coordination degree model^46^. However, the level of coordination effectively reflects the interdependence of sports tourism^47–49^. The procedures for determining the degree of coupling coordination are as follows:

First, calculation of coupling degree.

6 $$C=\sqrt{\frac{{U}_{1}{U}_{2}}{{\left(\frac{{U}_{1}+{U}_{2}}{2}\right)}^{2}}}$$

Second, calculation of overall evaluation index.

7 $$\mathrm{T}={\alpha U}_{1}+{\beta U}_{2}$$

In the above equation, α and β are the weight coefficients of sports and tourism subsystems, respectively, and each is taken as 0.5.

Third, calculating the coupling coordination.

8 $$\mathrm{D}=\sqrt{C\times T}$$

Fourth, calculation of relative development.

9 $$\mathrm{E}=\frac{{U}_{1}}{{U}_{2}}$$

The level of coupling cooperation between the sports and tourism industries in the Yellow River Basin was categorized and defined using a combination of academic studies^50^, as shown in Table 1.

**Table 1:** Classification and description of coupling coordination level.

D	Level stage	Description	Coupling coordination state
(0, 0.1]	Budding stage	The connection between sports and tourism industries is sparse, showing a disorderly state of development	Extreme disorder
(0.1, 0.2]			Severe disorders
(0.2, 03]			Moderate disorder
(0.3, 0.4]	Start-up phase	The relationship between sports and tourism industry is relatively close, and the interaction is gradually strengthened	Mild disorders
(0.4, 0.5]			On the verge of disorder
(0.5, 0.6]			Barely coordinated
(0.6, 0.7]	Stabilization stage	The sports and tourism industries are closely connected and promote each other and develop in an orderly manner	Primary coordination
(0.7, 0.8]			Intermediate coordination
(0.8, 0.9]	Mature stage	Sports and tourism industries are interdependent with a high degree of dependence	Good coordination
(0.9, 1]			Quality coordination

The total scores for the sports industry and the tourism industry are represented by U1 and U2, respectively. If E < 1, it means that the sports business is developing rather slowly. If E≈1, the speed of development for both the athletic and tourism industries is constant. If E > 1, the sports industry has made significant progress.

### The data source

The data in this paper are all from the official data of the state and the statistical units of nine provinces and autonomous regions in the Yellow River Basin. It is mainly obtained from Statistical Yearbook of Shandong Province (2012–2021), Statistical Yearbook of Henan Province (2012–2021), Statistical Yearbook of Shanxi Province (2012–2021), Statistical Yearbook of Inner Mongolia Autonomous Region (2012–2021), Statistical Yearbook of Ningxia Autonomous Region (2012–2021), and Statistics of Shaanxi Province Yearbook (2012–2021), Statistical Yearbook of Sichuan Province (2012–2021), Statistical Yearbook of Gansu Province (2012–2021), Statistical Yearbook of Qinghai Province (2012–2021), Statistical Yearbook of China Sports (2012–2021), and Statistical Yearbook of China Tourism (2012–2018) ), China Cultural Relics and Tourism Statistical Yearbook (2019–2021), and official websites of provincial governments and sports bureaus in the Yellow River Basin, etc. Among these, this paper uses the linear interpolation method to augment the data that is either missing or not officially disclosed, which is practical for the subsequent systematic analysis.

### Construction of index system

In order to further explore the promoting and pulling effects of input factors on output performance, the input–output analysis approach is designed to transform the level of integration of the sports and tourism sectors into input factors and output performance framework^51–54^. An assessment index system was created to measure the input and output of the sports tourism industry. Based on the input–output structure of the sports tourism industry, the second level index of integrated development of sports tourism is created concurrently.

#### Input–output of sports industry

According to a thorough analysis of the industry's level of contribution and its effect on output performance, the number of sports venues, government funding allocated to sports and related industries, and the number of employees in the sports system are selected as the indicators to measure the capital capacity and human resources of the sports industry. Additionally, as the sports industry and scientific and technological services combine, the distance between the two is gradually widening. Knowledge, technology, and human capital have developed into crucial elements of the high-quality growth of the sports business and have made a sizable contribution to the expansion of the modern sports sector. Therefore, the quantitative index of individuals joining research institutions may reflect the scientific rationalization of the input variables into the sports business. In order to determine how successfully the sports industry system can deliver human resources for its clients, the choice of legal person units in the sports and related sectors is employed. It may also be used as one of the input indices to determine the size of the sports sector in the Yellow River basin. By using the statistical yearbook data of the provinces and regions in the Yellow River Basin and ensuring the availability and comparability of the data, the sales revenue of sports lotteries, the added value of the sports industry, and the development quality of athletes are chosen as the output performance indicators of the sports industry.

#### Input–output of tourism industry

According to the examination of input–output structure correlation features, the hospitality, entertainment, housing, and other related kinds of business development are what the tourism sector relies on the most. According to the current new development philosophy, achieving carbon peaking and carbon neutrality as well as promoting the emergence of an ecological society are additional requirements and missions of high-quality development. Therefore, the number of travel agents, the number of star hotels, the number of workers in the lodging and catering industry, and the number of A-level tourist attractions are included in the selection of input element indicators for the tourism industry. As a measure of output performance indicators, the tourism industry uses the number of stays to determine the number of domestic tourists coming from abroad, tourist foreign exchange revenue, domestic tourism income, and the number of stays because the individual benefit form is too subjective and difficult to use as an objective and quantitative reference for related research.

In conclusion, 16 s-level index systems are specifically designed based on the scientific rationality of the integrated evaluation index system of the sports and tourist industry, and the index attributes are all good indicators, as shown in Table 2.

**Table 2:** Evaluation index system of the integration of sports and tourism industry in the Yellow River Basin.

System	First level index	Weight	Second level index	Weight	Attribute
Sports industry *U*_1_	Inputs *A*_1_	0.6567	Number of legal persons in sports and related industries *X*_1_	0.1659	+
			Number of sports venues *X*_2_	0.1249	+
			Government financial appropriation for sports and related industries *X*_3_	0.1140	+
			Staff of scientific research institute *X*_4_	0.0885	+
			Number of employees in the sports system *X*_5_	0.1634	+
	Outputs *A*_2_	0.3433	Revenue from sports lottery sales *X*_6_	0.1099	+
			Added value of sports industry *X*_7_	0.1371	+
			Quality of athlete development *X*_8_	0.0963	+
Tourism industry *U*_2_	Inputs *B*_1_	0.6300	Number of travel agencies *Y*_1_	0.2298	+
			Number of star hotels *Y*_2_	0.1333	+
			Number of people employed in the accommodation and catering industry *Y*_3_	0.1018	+
			Number of A-level tourist attractions *Y*_4_	0.1651	+
	Outputs *B*_2_	0.3700	Domestic tourist arrivals *Y*_5_	0.1315	+
			Number of inbound overnight tourists *Y*_6_	0.0509	+
			Foreign exchange income from tourism *Y*_7_	0.0544	+
			Income from domestic tourism *Y*_8_	0.1330	+

## Measurement and coupling evaluation of sports and tourism industry in Yellow River Basin

### Analysis of measurement results of sports and tourism industry

Using the calculation formula described above, the measurement results of the overall level of development of the sports and tourism sector in the Yellow River Basin from 2011 to 2020 are calculated and shown in the following figure. Tables 3 and 4 examine the time series' evolution in accordance with the growth and changes at various stages.

**Table 3:** Comprehensive evaluation value of sports industry in Yellow River Basin.

Year	Shanxi	Inner Mongolia	Shandong	Henan	Sichuan	Shaanxi	Gansu	Qinghai	Ningxia	Average value
2011	0.0510	0.0126	0.0600	0.0422	0.0371	0.0389	0.1100	0.0340	0.0803	0.0518
2012	0.0675	0.1226	0.0724	0.1198	0.0777	0.0571	0.1155	0.1188	0.1191	0.0967
2013	0.1675	0.2326	0.1448	0.1614	0.1523	0.1271	0.2001	0.1752	0.1509	0.1680
2014	0.2094	0.3081	0.2036	0.1913	0.1746	0.2081	0.2906	0.3405	0.2758	0.2447
2015	0.2899	0.3575	0.2640	0.3022	0.2379	0.2374	0.3320	0.3226	0.2612	0.2894
2016	0.3842	0.4130	0.3181	0.3007	0.3662	0.4307	0.4057	0.5621	0.3876	0.3965
2017	0.4942	0.6066	0.4348	0.4281	0.4153	0.5063	0.5855	0.7453	0.5663	0.5314
2018	0.6348	0.6610	0.6585	0.7902	0.7385	0.7143	0.7577	0.8418	0.7658	0.7292
2019	0.8273	0.7912	0.8133	0.8907	0.8905	0.7675	0.8920	0.7536	0.8355	0.8291
2020	0.7334	0.8446	0.9445	0.8847	0.7591	0.7810	0.8931	0.8578	0.8906	0.8432

**Table 4:** Comprehensive evaluation value of tourism industry in the Yellow River Basin.

Year	Shanxi	Inner Mongolia	Shandong	Henan	Sichuan	Shaanxi	Gansu	Qinghai	Ningxia	Average value
2011	0.0730	0.2986	0.2206	0.2326	0.2124	0.3342	0.1462	0.0840	0.2004	0.2002
2012	0.1948	0.3802	0.2683	0.2574	0.2445	0.3503	0.2659	0.1533	0.2018	0.2574
2013	0.1858	0.4675	0.3065	0.2988	0.3098	0.3655	0.2809	0.2240	0.2159	0.2950
2014	0.2318	0.4822	0.3131	0.2395	0.2717	0.2675	0.2239	0.3064	0.2571	0.2881
2015	0.3876	0.5315	0.4252	0.3769	0.3446	0.3333	0.2246	0.3870	0.2855	0.3662
2016	0.4237	0.5195	0.5195	0.5256	0.3750	0.3046	0.3160	0.3802	0.3818	0.4162
2017	0.5131	0.6899	0.6024	0.5713	0.4514	0.4942	0.4308	0.5568	0.5133	0.5359
2018	0.6690	0.6926	0.7305	0.6370	0.5730	0.6658	0.5246	0.6220	0.5816	0.6329
2019	0.8073	0.7432	0.7770	0.7958	0.7869	0.7463	0.7889	0.8360	0.7798	0.7846
2020	0.7352	0.6643	0.6139	0.7184	0.6913	0.5933	0.5993	0.6846	0.5568	0.6508

#### Analysis of comprehensive level measurement result of sports industry

The overall level of development of the Yellow River Basin sports industry has significantly risen over the last ten years, measured by the mean value. The comprehensive index's average value increased from 0.0518 in 2011 to 0.8432 in 2020, with an average annual growth rate of 36.34%. Standard deviation-wise, the Yellow River Basin sports industry's standard deviation from 2011 to 2015 is 0.0885, and the standard deviation of the entire sports industry from 2016 to 2020 is 0.1748. These figures show a little degree of dispersion in the overall growth of the sports industry during the past ten years, but over the previous five years, the standard deviation of the sports business has slightly increased. It illustrates the widening gap in regional development. As a result, it is possible to say that there are two stages to the level of development change. First, the Yellow River Basin's sports industry's capacity for development from 2011 to 2015 is comparatively weak, and the input and output effects of various indicators are obviously insufficient. This is largely because of the region's sluggish infrastructure development and low levels of public awareness of sports consumption. Second, from 2016 to 2020, the Yellow River Basin's sports industry's overall development level exhibited a tendency of medium–high development. The Second National Youth Games in Shanxi Province in 2019 and the 14th National Games in Shaanxi Province in 2021 are two major contributing factors, as are the implementation of pertinent national sports industry regulations. It increased the level of growth of the Yellow River Basin sports sector during the bidding, organizing, and holding processes and promoted the high-quality integration of sports tourism in the Yellow River basin. In the Yellow River Basin, Shandong's comprehensive level of the sports sector expanded after 2016 at a rate that was considerably faster than that of the other provinces and regions. Second, nodes of accelerated development occurred in 2016 and 2018, and the comprehensive level of the sports industry in Ningxia, Gansu, and Henan was also noticeably higher than the average level of the Yellow River basin. Together with Shandong Province, they make up the top tier of the Yellow River Basin's overall sports industry development level and perform exceptionally well at the development level. The development level of the sports industry in Qinghai and Inner Mongolia, which are included in the second tier, is comparable to the Yellow River basin average level and is expected to reach 0.84 horizontal line by 2020, indicating a relatively uniform performance. The Yellow River basin of the sports industry development level of regional differences further highlights the situation has not been obviously alleviated, backward development level gradually. According to change growth level of the sports industry development in the Yellow River, the third tier included in Shanxi, Sichuan, and Shaanxi, its development level is lower than the average level in the Yellow River, and the first echelon development level differences.

#### Analysis of comprehensive level measurement result of tourism industry

From 2011 to 2020, the average value shows a steady upward trend in the total degree of development of the Yellow River Basin tourism industry. The comprehensive index's average value will rise from 0.2002 in 2011 to 0.6508 in 2020 with an average annual growth rate of 13.99%. The Yellow River Basin's tourism industry's comprehensive level from 2011 to 2015 had a standard deviation of 0.0540, and its comprehensive level from 2016 to 2020 had a standard deviation of 0.1229, which shows that the industry's development level has shown a strong situation over the past five years. The development stage can be classified into three different levels from the standpoint of complete development level. First, while the general degree of development for the tourism sector in the Yellow River Basin from 2011 to 2014 was consistent, the development momentum was not immediately apparent. The lack of market awareness is mostly caused by the restricted development of tourism resources in tourist sites. The effectiveness and quality of tourism-related goods and services are low, making it impossible to satisfy consumers' high demands and causing the industry to merely see a quantitative increase in its output. Second, from 2015 to 2017, the Yellow River Basin's tourism industry experienced a remarkable improvement in its overall degree of development. Because of the then-rapid economic growth, which changed the structure of the tourist industry and actively supported the integration of sports tourism in the Yellow River Basin, this is the case. Third, the COVID-19 epidemic's effects on the provincial tourism market, which resulted in a significant drop in visitors and income, caused the comprehensive development level of the tourism industry in the Yellow River Basin to first rise and then slightly decline between 2018 and 2020. From the perspective of sub-regions, the comprehensive development level of the tourism industry in Shandong and Henan is firmly in the first echelon, and the development trend of the two provinces is similar. Both of them showed strong development momentum in 2016 and reached the peak in 2019. The overall development situation strongly leads the comprehensive level of the tourism industry in the Yellow River Basin, which is a star development region. Inner Mongolia and Shaanxi are categorized into the second echelon of the comprehensive development level of the tourism industry in the Yellow River Basin because they are all close to the average line in terms of the evaluation and analysis of the average development level of the industry in the Yellow River basin. The third tier includes Sichuan, Shanxi, Qinghai, Ningxia, and Gansu. It is important to note that even though the five provinces and regions have similar development situations, they are all below the Yellow River basin's average level and the overall development situation is not showing signs of strong acceleration, which will further widen the gap with the first tier. Further evidence that the level of the region's tourism industry is behind implies that this echelon comprises 55.6% of the provinces in the Yellow River basin. Given the rich regional resources, the third echelon's growth rate generally lacks velocity, which also demonstrates the urgent need for industrial transformation and digitization in the western Yellow River basin region.

### Evaluation of coupling coordination between sports and tourism industry

**Figure 2:**
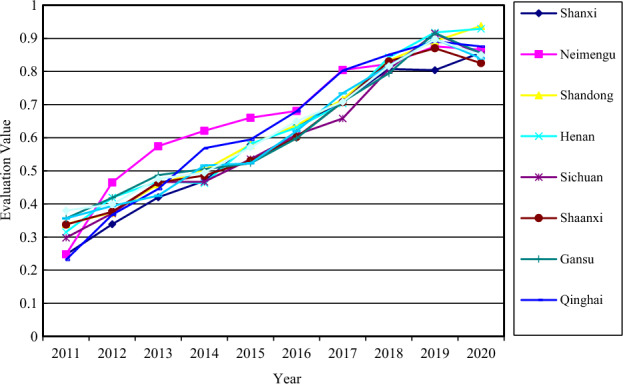
Dynamic evolution of coupling coordination degree of sports and tourism industry in the Yellow River Basin.

According to the calculation method of coupling coordination degree model, the coupling degree (C), coordination degree (D) and relative development degree (E) of the sports and tourism industry in the Yellow River Basin from 2011 to 2020 were obtained^55^, and Table 5 was made according to the coupling coordination index and the classification standard of coupling evaluation type. In order to evaluate the coupling and coordination development of sports tourism in the Yellow River Basin from 2011 to 2020 objectively, a dynamic evolution chart of the coupling coordination between the sports and tourism industry of each province in the Yellow River Basin from 2011 to 2020 was also created (Fig. 2).

**Table 5:** Coupling evaluation results of sports tourism in the Yellow River Basin.

Year	C	D	E	Coupling coordination state
2011	0.9359	0.3801	0.4790	Mild disorder
2012	0.9211	0.3989	0.4395	Mild disorder
2013	0.9924	0.4757	0.7813	On the verge of disorder
2014	0.9950	0.4944	1.2228	On the verge of disorder
2015	0.9996	0.5710	1.0603	Barely coordination
2016	0.9999	0.6494	1.0311	Primary coordination
2017	0.9995	0.7081	1.0675	Intermediate coordinate
2018	0.9983	0.8158	1.1229	Good coordination
2019	0.9983	0.8963	1.1234	Good coordination
2020	0.9740	0.8502	1.5855	Good coordination

According to Table 5, the Yellow River Basin's sports and tourist sector will have an increase in relative development degree (E) from 2011 to 2020. The E value for the period 2011 to 2013 is (0.4, 1), demonstrating that the Yellow River Basin's tourism sector is currently more developed than the sports sector. Consequently, the E value (1, 1.6) from 2014 to 2020 shows that the sports industry is developing at a leading rate and that its growth is about in line with that of the tourism sector. The aforementioned patterns show that the Yellow River Basin's tourism sector has a solid base. The tourism sector's all-encompassing capabilities can successfully compensate for the sports industry's lack of resources in the early stages of sports tourism integration. The ongoing hosting of major sporting events over the time period has fostered the development of infrastructure and comprehensive service level, and the overall level of the sports industry has been significantly raised as a result of the acceleration of the integration process of sports tourism. In order to achieve high quality development in the new stage of development, the sports tourism industry must focus on the management and protection of natural resources as well as improve the quality and efficiency of development. In light of this, the Yellow River Basin's current, mainly synchronous development of the sports tourism industry is commensurate with current development trends and needs.

#### Temporal characteristics of coupling coordination degree

Table 5 and Fig. 2 show that over the past ten years, there has been an overall increase in the coupling coordination development level of the sports and tourism industries in the Yellow River Basin. The coupling coordination degree varied between 0.4 and 0.8 in 2011 and 2012 before steadily rising in 2013—but especially in 2018–2020, when it exceeded 0.8. Additionally, the Yellow River Basin's sports and tourist business underwent a process of mild -- near imbalance, scarcely -- primary -- medium -- good coordination, and the coupling coordination connection gradually improved from 2011 to 2020. The average score for the decade from 2011 to 2020 for the degree of coupling coordination between the sports and tourism sectors is 0.6240, which is in the primary coordination level of coupling and coordinated development. Based on temporal factors, the degree of connection between the sports and tourism industries is divided into two stages: the coupling coordination degree increased during the initial phase (2011–2014) from 0.3801 to 0.4944. The quality of industrial economic development, however, remained low and had not yet switched to an interactive form. The interaction link was becoming increasingly stronger, and the coordinating relationship between the two industries was at this point fairly close. This is because the Chinese sports sector has grown slowly, a sizeable percentage is still devoted to the production of conventional sports, and the industry's potential has not yet been completely realized. Additionally, the tourism industry suffered from declining service standards at five-star hotels as well as at places of culture and entertainment during this time, which to some extent impeded the coordinated expansion of the sports industry and the tourism sector. The level of coupling coordination has increased throughout the steady stage (2015–2017). After the industrial integration's downturn time of optimization and adjustment, the new stage's positive development momentum has been sparked, and the two industries have achieved mutual promotion and orderly development. The creation of sports tourism, sports health, and other services served as a critical pillar for the industry's scale growth, and the sports industry had exponential growth during this time, mostly as a result of market-oriented reform and industrial policies. The mature stage (2018–2020) is defined by high-speed growth to medium–high-speed growth, broad development to the stage of high quality and quality enhancement of the industrial integration structure, and achieving a strong coordination level. The relationship between coupling and coordination is interdependent and highly dependent. In 2020, coupling coordination will be 0.8502. Additionally, it benefits from the enforcement of essential laws and the expansion of the sports tourism industry. In particular, the high-quality development plan has supported the modernization of the sports and tourism industries' industrial structures, resulting in a more sophisticated, logical, and environmentally friendly industrial structure. As a result, it will respond to the increased demand for sports tourism from locals by deepening the coupling and coordinating the growth of sports tourism.

#### Spatial characteristics of coupling coordination degree

The data source for further research into the regional development state of the coupling coordination between the sports and tourism industries in the Yellow River Basin was chosen to be the coupling coordination degree of 2011, 2014, 2017, and 2020. The spatial difference map was subsequently produced using ArcGIS software, allowing for more research on the development principles and regional traits.

**Figure 3:**
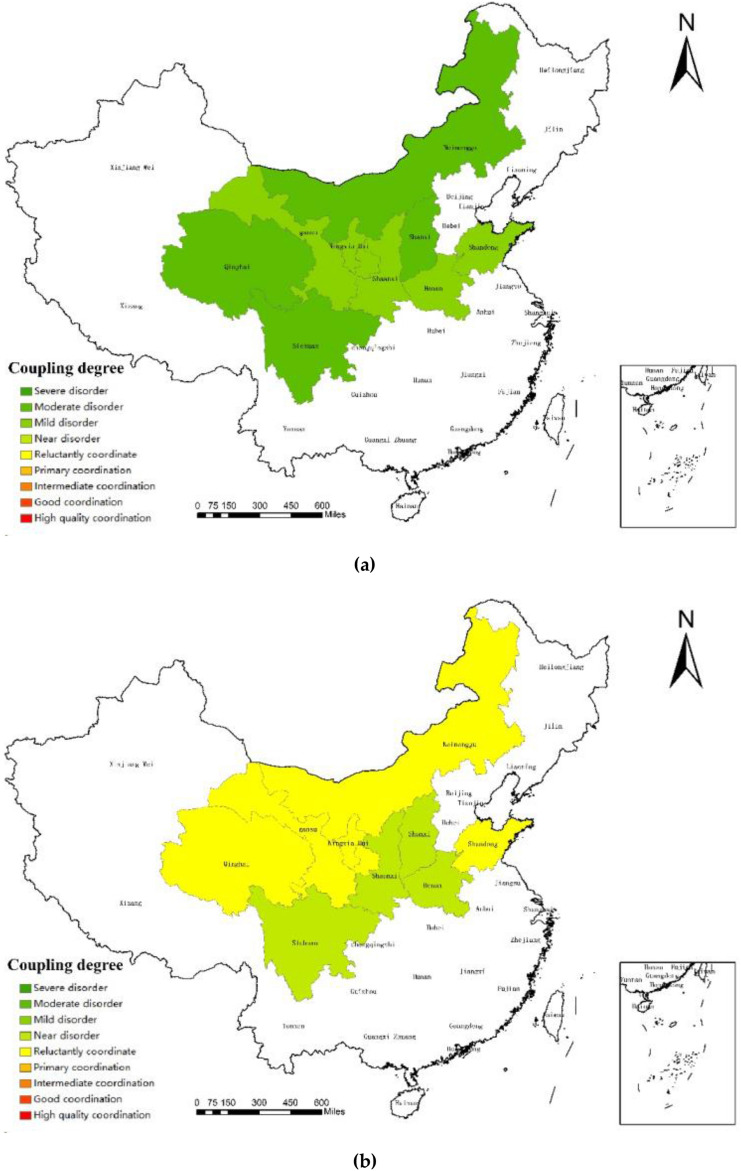

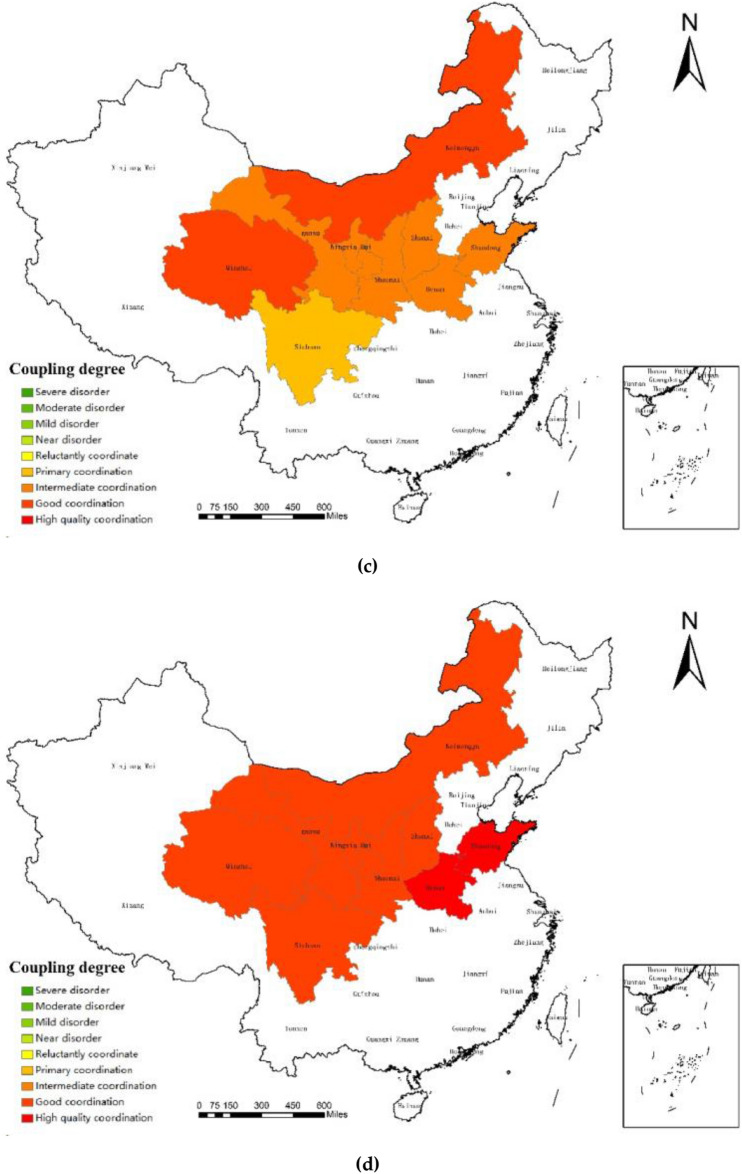
Spatial distribution of sports tourism coupling coordination degree in the Yellow River Basin in 2011**(a)**, 2014**(b)**, 2017**(c)** and 2020**(d)**. Source: Based on the standard map service website of the Ministry of Natural Resources, the approval number is GS (2020)4630. The base map boundary is not modified.

The Yellow River Basin has experienced regional differences in the degree of sports and tourist industry coupling cooperation over the past ten years, as shown in Fig. 3. The top, middle, and bottom reaches all clearly demonstrate the evolution law of "mature"—"backward"—"mature": (1) In the lower Yellow River Basin, Shandong and Henan's coordination level has mostly been in a steady development stage over the past ten years, and the growth trend of the sports and tourism industries has been quickening. Due to the support of its strong province economic development, the enormous population driving the consuming market, and the abundance of resources for sporting events, Shandong has the strongest coupling coordination level among them and will reach the mature stage in 2020. These regions also have abundant sports tourism resources and easy access to transportation, which is helpful for the coordinated growth of the local sports tourism business. (2) Shaanxi Province, which was in the early stage from 2011 to 2015 and initially reached the primary coordination state in 2016, has the highest coupling coordination degree in the middle reaches of the Yellow River. However, Shanxi has long maintained a moderate to almost balanced condition, and there is little correlation between the sports and tourism sectors there. This is partly due to the regions' poor economic development, inland location, challenging transportation, and relatively underdeveloped industrial level. Additionally, it draws attention to the practical problems brought on by sports participation and the underuse of tourism-related resources. (3) In the upper reaches of the Yellow River, Inner Mongolia, Ningxia, Gansu, Sichuan, and Qinghai provinces' coupling coordination levels are relatively close, regional development disparities are negligible, and the map's "block" and "strip" agglomeration shapes are clearly created. The highest degree of comprehensive coupling coordination is found in Inner Mongolia, with the other four provinces' degrees generally falling between the average and intermediate ranges for extended periods of time. This indicates that the relationship between the sports and tourism industries is relatively close, and that the rising state is developing. The higher portions of the Yellow River have abundant tourist resources, which is primarily to blame for this. The enormous amount of visitors and their purchasing power drive the local economy's level of development and support the linked and coordinated development level of the sports tourism sector.

Last but not least, from the standpoint of input–output theory, the quantity of input elements and the quality of output performance are what largely influence the amount of coupled coordinated growth of sports tourism in the Yellow River Basin. The Yellow River Basin's input factor indices have increased significantly year after year over the past ten years, and the industrial transition has seen some early success. There has been no discernible trend shift in the level of coordinated development as a result of changes in the industrial added value and tourism income in the output performance, which have increased and decreased, respectively. Among them, the variations in the output performance of different subdivisions also show how, over the past ten years, the integrated structure of sports tourism in the Yellow River Basin has undergone dynamic adjustment.

#### Kernel density estimation

In statistics, kernel density estimation is typically based on small samples to infer the distribution state of the overall data^56,57^. The calculation result of kernel density estimation is the probability density function estimation of the sample, and the result form is an uninterrupted density curve^58,59^. The kernel density estimation approach is used to evaluate the distribution dynamics and evolution characteristics of the coupling coordination level between the sports and tourism industries in the Yellow River Basin, and the calculation method is provided in Eq. (10):

10 $$\mathrm{P}\left(x\right)=\frac{1}{N}\sum_{k=1}^{N}\frac{1}{h}K\left(\frac{x-{x}_{k}}{h}\right)$$

where P(x) is the integrated development index of sports and tourism in the Yellow River Basin, namely, the coupling coordination degree (D); N is the number of observations; N is the bandwidth. K is the kernel function; X is the mean number of observations. Since Gaussian kernel functions adopted in this paper^60^, the calculation formula is Eq. (11).

11 $$\mathrm{K}\left(x\right)=\frac{1}{\sqrt{2\pi }}exp\left(-\frac{{x}^{2}}{2}\right)$$

**Figure 4:**
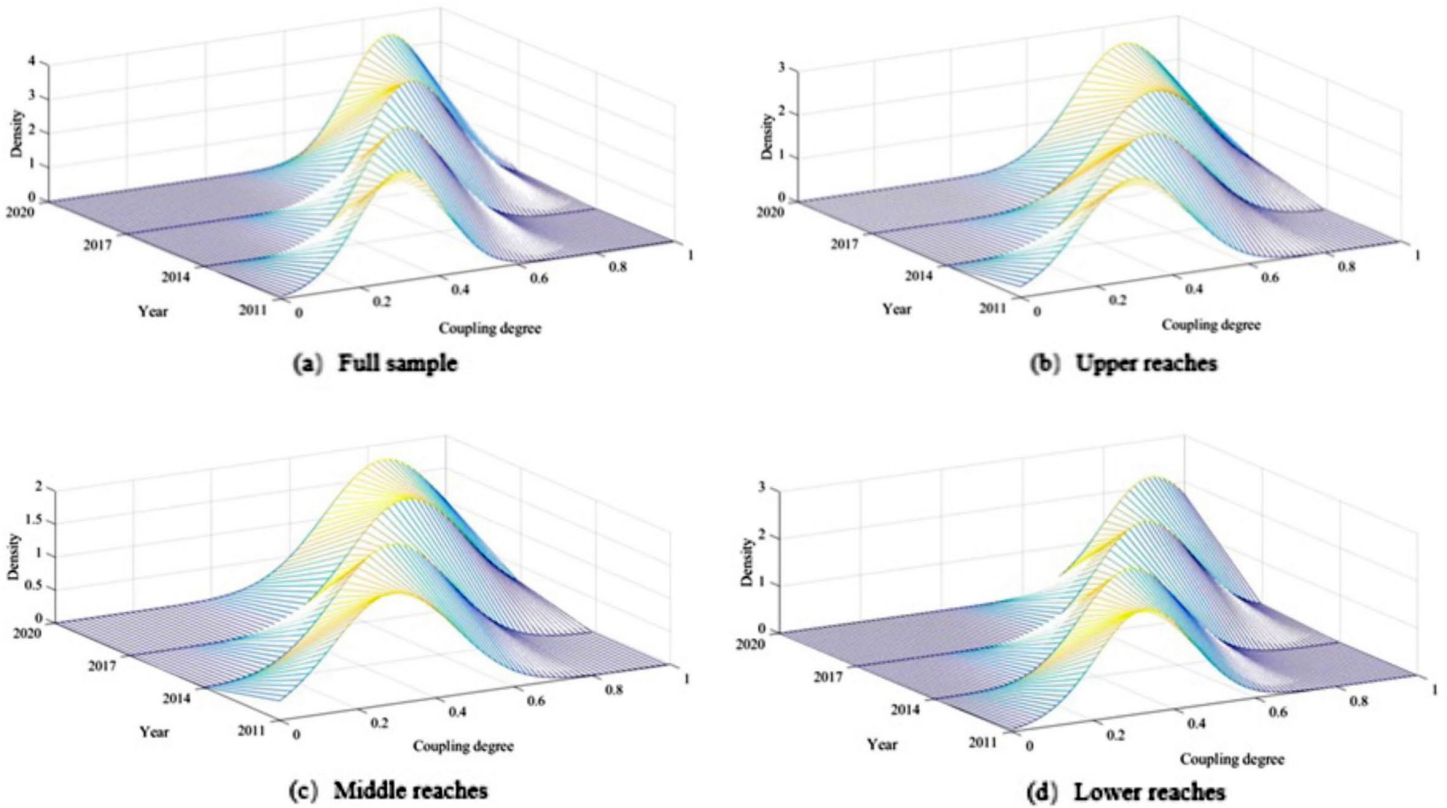
Kernel density curves of coupling coordination degree in different regions in representative years.

Four representative years—2011, 2014, 2017, and 2020—were used to create the kernel density curve of the coupling coordination degree of the sports and tourism industries in the Yellow River Basin. The Yellow River basin's four plates—the entire region, the upstream, the center, and the downstream—were used to illustrate the ductility, polarization, and distribution of the coupling coordination level kernel density curve. There are absolute differences in the coupling coordination degree in the Yellow River Basin, as shown in Fig. 4a, but it generally exhibits a trend of contracting first and then expanding. From 2011 to 2020, the peak height of the wave increases year by year, the width of the curve increases, the right tail is elongated, and the ductility is broadened. In terms of the wave peak's position, the wave peak gradually shifted to the right with each passing year, showing that the Yellow River basin's coupling coordination level was improving. The lack of a multi-peak phenomenon in terms of the number of primary peaks suggests that there is no multi-polarization trend. From Fig. 4b, c: found in the Yellow River upstream section close to the middle period of nuclear density curve and representative of wave height, the curve shape gradually narrows, reduces right trailing phenomenon, indicating that the upstream and middle coupling coordination level of regional differences is in a narrow situation and that development level is generally balanced; density curve and wave peak move to the right, indicating that the coupling coordination is getting better. There is no multi-peak curve and a clear single peak, which excludes the existence of the multi-polarization phenomenon. The position of the wave peak is continuously moving to the right in Fig. 4d, which suggests that the downstream segment's coupling coordination level is typically increasing. The coupling coordination level in the downstream zones was clearly different but showed a trend toward narrowing, indicating that there was no multi-polarization phenomenon. From the perspective of curve form, the curve shifted from "flat" to "narrow" from 2011 to 2020.

## Correlation measurement and driving factors analysis of sports and tourism industry in Yellow River Basin

### Index selection of correlation degree between sports and tourism industry

Given that the sports tourism sector is a recent development in the economy, a number of factors affect the degree of integration. In light of relevant academic studies, the sample size for the grey correlation study was determined^61,62^. The author increased the output value of tertiary industry to GDP (TER) on the basis of integrating the 16 evaluation factors in Table 2 in order to evaluate the agglomeration scale effect of the service industry in the Yellow River Basin. The number of granted invention patents (IPA), which measures the level of investment in scientific and technological innovation in the Yellow River basin, the total number of employment in the sports system, and the total retail sales of consumer goods (CON), which reflect the consumption level of residents in the Yellow River basin Additionally, the total number of workers in the Yellow River Basin (LAB) to reflect employability and other distinctive data supplied by the Yellow River Basin sports and tourism business.

### Measurement of correlation degree between sports and tourism industry

The main goal of grey correlation analysis is to compare changes in the sequence curves of the research objects to determine how closely the two correlate. The closeness will be stronger if the change is closer and weaker if the change is farther away^63,64^. The ability of grey correlation analysis to make accurate calculations and descriptions even in the presence of limited information data is one of its most notable features. Part of the material is lacking as a result of the Yellow River Basin's slow integration of sports tourism and insufficient development, which is in line with the application criteria of the grey theory system^65,66^. The precise degree of coupling between each parameter and sports tourism in the Yellow River basin can thus be measured using the grey correlation analysis method. The calculation process is as follows:

First, determine the reference and comparison data columns. The reference data column is usually the data column that can fully represent the characteristics of the system trend. According to the evaluation purpose, the coupling coordination degree of the sports and tourism industry in the Yellow River basin is selected as the reference sequence, denoted as $\mathrm{Y}=\left\{Y\left(k\right) \vert \mathrm{k}=0\right\}$, the comparison sequence is denoted as ${X}_{i}=\left\{{X}_{i}\left(k\right) \vert \mathrm{k}=\mathrm{1,2},\dots ,\mathrm{n}\right\},\left(i =\mathrm{1,2},\dots ,15\right).$

Second, initial value method was used to normalize the index data.

12 $${X}_{i}^{\widehat{\,} }=\frac{{X}_{i}}{{X}_{i1}}\left(i={1,2},\dots ,m \right)$$

Third, the absolute difference between the values of each evaluation index and other characteristic variables and the coupling coordination degree is calculated.

13 $${\Delta }_{i}\left(k\right)= \vert {X}_{0}^{\widehat{\,}}\left(k\right)-{X}_{i}^{\widehat{\,}}\left(k\right)\vert \left(i=\mathrm{1,2},\dots ,m;k=\mathrm{1,2},\dots ,n\right)$$

Fourth, determine the two-level minimum difference and the two-level maximum difference.

14 $${min}_{i=1}^{n}{max}_{k=1}^{m}\vert {X}_{0}\left(k\right)-{X}_{i}\left(k\right) \vert$$

15 $${max}_{i=1}^{n}{max}_{k=1}^{m} \vert{X}_{0}\left(k\right)-{X}_{i}\left(k\right)\vert$$

Fifth, calculate the correlation coefficient.

16 $${\xi }_{i}\left(k\right)\frac{{min}_{i}{min}_{k}\vert{X}_{0}\left(k\right)-{X}_{i}\left(k\right)\vert+\rho \bullet {max}_{i}{max}_{k}\vert{X}_{0}\left(k\right)-{X}_{i}\left(k\right)\vert}{\vert{X}_{0}\left(k\right)-{X}_{i}\left(k\right)\vert+\rho \bullet {max}_{i}{max}_{k}\vert{X}_{0}\left(k\right)-{X}_{i}\left(k\right)\vert} \left(k=\mathrm{1,2},\dots ,n\right)$$

The correlation coefficients corresponding to the values of each evaluation index and other characteristic variables and the coupling coordination degree are calculated separately, where ρ is the discrimination coefficient, and the value is taken within (0, 1), if the value of ρ is larger, the correlation coefficients are less different from each other, and the discrimination effect is better^67^, usually the value of ρ is taken as 0.5.

Sixth, calculate the correlation.

17 $${r}_{0i}=\frac{1}{m}{\sum }_{i=1}^{m}{\xi }_{i}\left(k\right) \left(k=\mathrm{1,2},\dots ,m\right)$$

The correlation degree generally takes values between (0, 1), and the closer the result is to 1, the higher the degree of correlation, and vice versa, the weaker the correlation effect. The specific correlation degree level is divided as shown in Table 6.

**Table 6:** Classification of relevance level.

${r}_{0i}$	(0.95, 1]	(0.85, 0.95]	(0.65, 0.85]	(0.45, 0.65]	(0, 45]
Level	Extremely strong correlation	Strong correlation	Medium correlation	Weaker correlation	Extremely weak correlation

### Ranking and analysis of correlation degree between sports and tourism industry

The findings indicate that there is a substantial coupling correlation effect between each evaluation index and sports tourism in the Yellow River Basin. A further indication that the 20 variables have a strong pulling and promoting effect on sports tourism integration is the fact that the correlation degree for 18 of the 20 indexes is better than 0.8, and none is lower than 0.4, according to Table 7.

**Table 7:** Ranking of sports tourism coupling grey correlation degree in the Yellow River Basin.

No.	System index of sports industry	${r}_{0i}$	System index of tourism industry	${r}_{0i}$	Characteristic variables	${r}_{0i}$
1	Government financial allocations for sports and related industries	0.9807	Number of A-level tourist attractions	0.9768	CON	0.9929
2	Staff of scientific research institute	0.9752	Domestic tourist arrivals	0.9589	TER	0.9472
3	Quality of Athlete Development	0.9632	Number of travel agencies	0.9493	IPA	0.7397
4	Added value of sports industry	0.9383	Number of people employed in the accommodation and catering industry	0.9286	LAB	0.4544
5	Number of legal persons in sports and related industries	0.9326	Number of inbound overnight tourists	0.9284		
6	Revenue from sports lottery sales	0.9110	Foreign exchange income from tourism	0.9218		
7	Number of sports venues	0.8952	Number of star Hotels	0.9068		
8	Number of employees in the sports system	0.8884	Income from domestic tourism	0.8922		

#### Analysis of extremely strong correlation indicators

Among the 20 indicator variables there are six extremely strong correlation indicators are CON (0.9929), government financial allocation for sports and related industries (0.9807), number of A-class tourist attractions (0.9768), personnel of research institutes (0.9752), quality of athlete development (0.9632) and number of domestic tourists (0.9589), indicating that the level of consumption, investment capacity and infrastructure development are most strongly associated with the sports tourism industry. The most logical reflection of the input components and performance of sports tourist activities from the standpoint of capital capacity is the government's financial allocation and CON of sports and related businesses. A-class tourist attractions, which are the fundamental capacity of people congregating to participate in the consumption of sports tourism activities, indicate the level of infrastructure supply in the process of people's participation in sports tourism from the standpoint of infrastructure construction. The results of scientific research and innovation also have a clear pulling impact on how sports tourism is coupled. The supply-side reform of the sports tourism industry is still being promoted as the social and economic level enters the stage of high-quality development, and naturally, scientific and technological innovation becomes the primary influencing factor of high-quality development of the sports tourism industry.

#### Analysis of strong correlation indicators

The strong correlation indicators derived from the measurement results are the number of travel agencies (0.9493), TER (0.9472), added value of sports industry (0.9383), number of legal persons in sports and related industries (0.9326), number of employees in the accommodation and catering industry (0.9286), number of inbound overnight tourism (0.9284), foreign exchange earnings from tourism (0.9218) , sports lottery sales revenue (0.9110), the number of star-rated hotels (0.9068), the number of sports venues (0.8952), domestic tourism revenue (0.8922), and the number of employees in the sports system (0.8884). The bulk of the aforementioned 12 indicators are categorized under the headings of revenue and human resources in the sports tourism industry. The Yellow River Basin's sports tourism sector has just recently started to grow from the perspective of industry revenue, so its revenue situation and market share scale are less dependent on the level of integration of sports tourism. The sports tourism sector may offer a lot of employment prospects from a human resources point of view, which is important for attracting top talent and supporting regional economic and social growth. However, there is a significant gap with the more developed provinces and cities in this sector. The rapid development of sports tourism offers significant advantages for the expansion of tourist spending as well as the improvement and modernization of the tertiary sector structure, in addition to bringing in more domestic and foreign tourists. As a result, the integrated growth of sports tourism can not only bring more distinctive and high-quality service products, greatly satisfy consumer demand for good consumption, and thereby increase the industry's revenue, but it can also bring significant revenue, which can be used to fully upgrade the environment's infrastructure for sports tourism and increase consumer enthusiasm.

#### Medium and weak correlation analysis

The medium and weak correlation indexes between (0.45, 0.85] are IPA (0.7397) and LAB (0.4544), both of which are characteristic variables. It demonstrates that, from the perspective of invention patents granted, IPA provides the most intuitive reflection of the knowledge capacity output of sports tourism activities, while the Yellow River Basin city cluster urgently requires the introduction of high-level talent due to the current state of industrial transformation, as evidenced by its low ranking in the correlation ranking. From the perspective of market development, the LAB indicators have little impact on how sports tourism is integrated. Thus, it is found that improving employability through market diversification development is the way of the future, and that improving industry digitization, business viability, job opportunities, and the city cluster's ability to absorb applied and research-oriented talents are crucial supports for the high-quality development of sports tourism.

### Analysis of the driving factors of sports tourism integration development

Integrating sports and tourism makes it possible to share and complement basic resources, and combining social and natural resources gives the growth of the social economy a boost^68^. At the same time, the deep integration of sports tourism lies in the exogenous drive of government policy orientation^69,70^ and the compatibility of sports tourism resources. The endogenous drive of personalized development of sports tourism products to promote market demand^71^, enterprise transformation and cultivation^72^, and industrial value chain decomposition and reorganization^73,74^. In light of this, this research investigates, using the findings of a grey correlation analysis, the driving forces behind the integrated development of sports tourism in the Yellow River Basin.

#### Government macro policies promote the integration of sports tourism

In order to achieve the goal of industrial value-added, the current government has proposed new guidelines for the integrated development of the sports tourism sector, one of which is to concentrate on the qualities of sporting events that draw spectators. The Yellow River Basin sports and tourist industry has advanced the critical idea of hastening the integration of sports tourism development in a timely way, as has been done in other economic and social fields. It is necessary to take advantage of the market opportunity presented by the mass tourism era and the upgrading of sports tourism consumption in order to realize the unified promotion of the integration of the sports tourism industry in the Yellow River Basin. Integration of sports and tourism answers to current demands for development, encourages businesses to innovate products and services more quickly, and supports the long-term success of associated businesses.

#### Resource sharing lays the foundation for sports tourism integration

**Figure 5:**
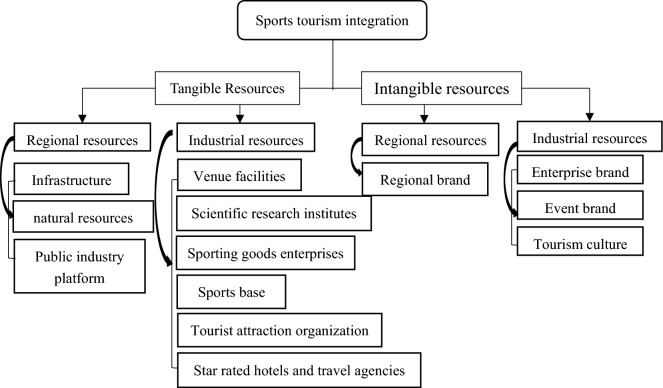
Sports tourism industry integration resource sharing chart.

There are many places where sports and tourism share resources, as illustrated in Fig. 5. Thousands of tourists are drawn to engage in consumer activities each year by facilities like ski resorts, sports museums, and club halls of fame, which are examples of tangible resources. Regarding intangible assets, IP of prestigious international competitions like the Olympic Games and the World Cup every four years even draws a sizable number of sports lovers to watch and visit the host country. Consider the Winter Olympics in Beijing in 2022. There will likely be 340 million snow and ice tourists in China. The resources that sport and tourism each have are advantageous, and they can be shared simultaneously. Sports tourism offers rich consumer goods with distinctive qualities in terms of regional resources, from the sporting traditions passed down by ethnic groups in China to the tourism resources of landform wonders. In terms of industrial resources, concrete resources like sports facilities and sports bases can be used as tourism products for viewing and experiencing, while concrete resources in the tourism industry, like scenic locations and scenic locations, can be used as locations to hold sporting events and enhance the impact of fitness activities.

#### Market demand activates the momentum of sports tourism integration

The market demand is the primary driver of the expansion of the sports tourism industry. As people's income levels usually rise, they gradually become less dependent on material items and grow more concerned with their quality of life, according to an assessment of the shifting trend in tourist demand. Traditional mono-type tourism material cannot quench the modern public's need for fitness, involvement, and competition, therefore suppliers must concentrate more on leisure travel, experiential travel, ecological travel, and other forms of travel. However, the fundamental characteristics of sports serve to support such reasonable objectives. Sports serve as a means of socialization, pressure release, and physical development. The current decline in tourist market consumption can be successfully reversed by integrating sports and tourism content. In order to suit the expectations of tourists, sports tourism is a new type of travel that places a strong emphasis on engagement, challenge, stimulation, and professionalism. It enables travelers to take in a range of grueling competitions while on the road and even participate in some of them themselves, such as cycling, skiing, swimming, mountain climbing, and rock climbing. Under the demand for memorable travel experiences, it will continue to support the high-quality expansion of the integration of sports and the tourist industry.

#### Enterprise transformation and growth cultivation release the vitality of sports tourism integration

The Yellow River Basin has recently been in a vital phase of industrial structure optimization and adjustment as well as green and sustainable business transformation. Supporting the development and transformation of sports tourism businesses is advantageous for maximizing the use of local resources in the Yellow River basin and fostering the steady economic expansion of the tertiary sector. In light of the vast sports tourism consumer market, businesses' emphasis on the benefits of product output also aids in the objective realization of sports tourism integration. Business owners can reduce production costs and inputs while also developing innovative new goods and business models based on the diverse, unique needs of customers by combining sports and tourist resources. For instance, the city marathon, which has gained popularity in recent years, might draw a sizable number of runners from both domestic and foreign countries to participate in the marathon. They will take part in other activities as well, such visiting nearby hotels and tourist places, in addition to having fun during the event. On the other hand, depending on the unique requirements of residents' families, the event organizer will also develop event products like parent–child runs and small runs, effectively expanding the enterprise revenue model. The incorporation of sports tourism increases the industrial development's commercial opportunities, areas for industrial growth, and areas for investment returns. It also reduces the capital risk associated with a single investment and operation, which increases the industrial development's sustainability.

#### Value chain decomposition and restructuring promote the upgrade of sports tourism integration

The breakdown and reorganization of the industrial value chain is a necessary prerequisite for the integration of sports and tourism. In order to promote market competitiveness, develop and optimize the core business processes of sports tourism companies, and lower management costs, sports tourism in the Yellow River Basin should systematically adhere to the features of the industrial chain. All areas of life are currently beginning to appreciate the economic benefits of sports and tourism, and in this favourable environment, the two industries' integration is expanding even further. This integration is being realized gradually from product to organization to market. Additionally, as the sports and tourist sectors continue to be integrated, it is important to better investigate each sector's benefits and work to identify the strategic intersections with its own value chain. The two industries are fully integrated into the production value chain as a result of industry penetration, extension, and rearrangement, which boosts business economic efficiency while achieving continuous chain structure improvement.

## Conclusions

### Conclusion

The linked measures and drivers of sports tourism in the Yellow River Basin from 2011–2020 are examined in this research using the entropy approach, coupled coordination degree model, kernel density estimation, and grey correlation analysis to achieve the following results.

First off, the Yellow River Basin's sports and tourist businesses have grown steadily and favorably over the past ten years. The first echelon of regional development is made up of Shandong, Henan, Ningxia, and Gansu; the second echelon is made up of Qinghai and Inner Mongolia; and the third echelon is made up of Sichuan, Shanxi, and Shaanxi. Shandong and Henan continue to be the top two in terms of the development of the tourism sector, followed by Inner Mongolia and Shaanxi, which are close behind. Sichuan, Shanxi, Qinghai, Ningxia, and Gansu, on the other hand, are at the bottom due to their poor capacity for development and unstable ecological conditions.

Second, the coupling coordination degree model states that over the past ten years, the sports tourism industry in the Yellow River Basin has evolved through six states: "mildly -- nearly dysfunctional, barely -- primary -- moderate -- good coordination," and generally demonstrates a trend of progress from the early stage to the stable stage and then to the mature stage. The spatial characteristics indicate the Yellow River basin's downstream > upstream > intermediate coupling coordination level development pattern. The kernel density estimation research shows that the Yellow River Basin's coupling coordination level has a significant regional variation and a rising trend. Regional differences, however, are not readily apparent from the examination of the upstream, midstream, and downstream modules, and the multi-polarization phenomena is not present.

Third, the construction of infrastructure and investment capability is the key factor in the development of sports tourism integrative, as shown by the grey correlation analysis of 20 variable coupling coordination degree of correlation calculation, which found that CON, sports and related industry government funding, a-class tourist scenic spot quantity index of the Yellow River basin, such as sports tourism integration correlation function, is extremely significant.

### Policy enlightenment

Based on the aforementioned findings, it is suggested that the deep integration of sports tourism in the Yellow River Basin because the growth of sports tourism integration in the Yellow River Basin started late and the process of sports tourist integration needs a certain amount of time to produce evident benefits.

1. The development levels of the top, middle, and lower portions of the Yellow River Basin are very diverse, according to the results of the coupled coordination measurement of sports tourism. In light of its unique characteristics, the Yellow River Basin should undertake varied development methods. For instance, to maintain the advantages of industrial development, the developed areas in the lower Yellow River should continue to optimize the industrial structure, increase the degree of industrial cooperation and agglomeration, and support the high-quality development of the sports tourism industry with the digital economy. The less developed regions in the Yellow River's middle and upper reaches should invest heavily in regionally specific sports tourism products, draw tourists to sporting events, build brands with regional appeal, improve industry-spatial correlation, and create an industrial regional linkage effect. Respond actively to the Yellow River Basin's strategy of ecological preservation and high-quality development, advance the coordinated agglomeration of the sports tourism industry through policy direction, establish economies of scale and range, lower marginal production costs, and foster the growth of small and medium-sized businesses.
2. The coordinated development trend of the several regions in the Yellow River Basin must be strengthened, according to the current national strategic orientation of the Yellow River Basin. In order for all departments to fully participate in the development of sports tourism and realize cross-regional information sharing, coordinated action, unified planning, and complementary advantages, it is important to strengthen the connections between the various regions in the Yellow River Basin and to expedite the establishment of cooperation mechanisms between sports, tourism, transportation, land, finance, and other departments. The coupling coordination mechanism of the sports tourism industry should be established and strengthened in order to achieve the highest market allocation, realize the high-quality development of the industry while operating within the constraints of resources and the environment, and increase the rate at which resources are utilized.
3. The output of innovation patents and population size have the lowest correlation coefficient, which suggests that the Yellow River Basin's innovation technology is still insufficient, according to the findings of grey correlation study. The efficiency and quality of sports tourism offerings must be increased as a result. And because the Yellow River Basin's current consumption level is relatively low, the output of high-end scientific and technological innovation is lower, and the professional ability of market players is weak, the ongoing cultivation of sports-related scientific and technological innovation is unquestionably a significant contributor to the holistic development of sports tourism. In order to produce and design new sports tourism consumption products with scientific and technological elements, such as smart wear, smart scenic spots, smart venues, virtual travel, etc., it is first necessary to integrate Internet resources and make full use of interactive technology, cloud computing technology, artificial intelligence technology, digital twin technology, etc. Second, actively investigate the sports tourism industry's digital innovation system to encourage it to boost effectiveness and quality. Currently, we must maintain a supply that is innovation-driven, gradually eliminate outdated manufacturing capability, and accomplish an all-around drive of the sports tourist industry chain, innovation chain, capital chain, and talent chain. This is due to the waning demographic dividend. Finally, it encourages the inclusion of sports tourism in university curricula in light of the talent gap in the market. Sports, tourism, management, geography, and other disciplines are all included in the course material so that students can build and operate sports tourism routes, sell their services as travel guides, and provide guidance for sporting events.

Simply put, the integration of sports and tourism is a dynamic evolution process of ongoing innovation and optimization between the two systems of the sports and tourism industries, which will be influenced by some external environment in the research. However, further in-depth analyses of spatial characteristics and comparisons of regional differences cannot be conducted in the Yellow River Basin due to the scarcity of data resources and the absence of policy implementation effects. The major study direction in the following stage is sports tourism, and we will continue to explore its effects while also improving the index evaluation system.

## Data Availability

The dataset used in this study is available from the corresponding author upon request.
